# Physical exercise for prevention of dementia (EPD) study: background, design and methods

**DOI:** 10.1186/s12889-019-7027-3

**Published:** 2019-05-29

**Authors:** Enzo Iuliano, Alessandra di Cagno, Adriana Cristofano, Antonella Angiolillo, Rita D’Aversa, Santina Ciccotelli, Graziamaria Corbi, Giovanni Fiorilli, Giuseppe Calcagno, Alfonso Di Costanzo, EPD Study Group, Giovanna Aquino, Vittorio Arcari, Lorenzo Buongusto, Giuseppina Cavallo, Mario Faraone, Nicola Ferrara, Mariella Filangieri, Melissa Fiscarelli, Sandro Iavarone, Francesca Iannetta, Stefano Moffa, Pasquale Mignogna, Giovannangelo Oriani, Federica Palombo, Tina Panichella, Silvia Pedata, Bruno Petti, Marilinda Spaziano, Maurizio Taglialatela, Roberto Valente

**Affiliations:** 1 Faculty of Psychology, eCampus university, Via Isimbardi 10, 22060 Novedrate, Italy; 2 Department of Medicine and Health Sciences, University of Molise, Via F. De Sanctis 1, 86100 Campobasso, Italy; 3 Department of Movement, Human and Health Sciences, University of Rome “Foro Italico, Piazza Lauro De Bosis 6, 00135 Rome, Italy; 4 Center for Research and Training in Medicine of Aging, Department of Medicine and Health Sciences, University of Molise, Via F. De Sanctis 1, 86100 Campobasso, Italy

**Keywords:** Mild cognitive impairment, Subjective memory complaints, Physical activity, Neuropsychological test, Aging population

## Abstract

**Background:**

Several observational studies have shown that exercise reduces the risk of cognitive decline; however, evidences from long-term, well-conducted, randomized controlled trials are scanty. The principal aim of this study is to verify whether a long-term program of multimodal supervised exercise improves the cognitive function and/or reduces the rate of cognitive decline in older adults at different degrees of risk for dementia.

**Methods/design:**

EPD is a parallel group, double-blind, randomized controlled trial. Community-dwelling volunteers aged 50 years or more are being recruited from different community centers and screened for eligibility. Enrolled subjects are being divided in 3 groups: a) without subjective or objective cognitive impairment, b) with subjective memory complaints, and c) with mild cognitive impairments. Participants in each group (at least 180) are being randomly assigned (1:1) to an experimental group, performing a supervised training including aerobic and resistance exercises of moderate/high intensity, or to a control group. Primary outcome will be 48-months changes in Mini Mental State Examinations. Secondary outcomes will be changes in several cognitive tests including a composite cognitive score. Time points will be at baseline, and at 6, 12, 24, 36 and 48 months. Statistical analysis will be done as intention to treat, complete case and mixed model analysis.

**Discussion:**

EPD is the first trial to examine the effects of a long exercise program (48 months) on cognitive performances. If successful, this trial may provide evidence for using long-term and multimodal exercise interventions for dementia prevention programs in the aging population.

**Trial registration:**

The study is registered at ClinicalTrials.gov with the code NCT02236416.

## Background

According with the report of the Alzheimer’s Disease International published in 2015, 46.8 million people are estimated to live with dementia worldwide, and this number might reach 131.5 million by 2050 [1]. Consequently, preventing and/or delaying cognitive impairment is a public health priority.

Alzheimer’s disease (AD) is the most common form of primary dementia and loss of episodic memory is often an early clinical symptom. The brain changes of AD may begin 20 or more years before symptoms appear [2], then identification of preclinical stages and preventive strategies to delay cognitive decline have become the focus of recent research. Several studies demonstrated that AD may be preceded by conditions known as subjective memory complaints (SMC) and/or mild cognitive impairment (MCI), that may be transitional stages between normal aging and dementia. SMC indicates a self-reported memory decline in the absence of pathological results on neuropsychological tests; it was estimated that elderly people with SMC have 2.7 times higher risk of developing dementia [3]. The estimated prevalence of MCI ranges from 10 to 20% with a rate of progression from MCI to dementia that variates from 5 to 20% per year depending on the examined population [4]. Furthermore, it has been calculated that if we could slow the progression to dementia in subjects at risk for just 12 months, in the 2050 we could have 9.2 million fewer cases of AD worldwide [5]. This delay in AD onset can represent a fundamental strategy, because nowadays AD is a fatal disease with no effective cure or treatment [6].

Despite the lack of therapies, several studies have shown that lifestyle modification and reduction of modifiable risk factor for AD offers a promising way of decreasing the risk of dementia [7, 8]. Physical inactivity is considered one of the seven main potentially modifiable risk factors for AD and explains approximately 13% (nearly 4.3 million) of AD cases worldwide [9]. However, some authors have reported that insufficient evidence exists to confirm the protective role of physical exercise in preventing AD onset [10]. The randomized controlled trial (RCT) investigating the effect of exercise on cognition did not provide definitive results due to the variation between study designs [11]. Two recent meta-analyses [12, 13] stated that the positive effects of exercise on cognition are driven by interventions that include aerobic exercise. Nevertheless, one of these two meta-analyses [13] concluded that methodological limitations of the included studies make the results interpretable with caution. Overall, the main limitations found in literature concerned the duration of the interventions that is usually lower than 12 months and the low standardization of the exercise protocol. Thus, well-designed RCT are recommendable and necessary [11, 12].

For these reasons, the aim of the present study is to verify if a 48-month program of supervised exercise reduces the rate of cognitive decline among older adults at risk of dementia. To the best of our knowledge, this RCT will represent the first study with such long duration.

## Methods

### Trial design

**Fig. 1:**
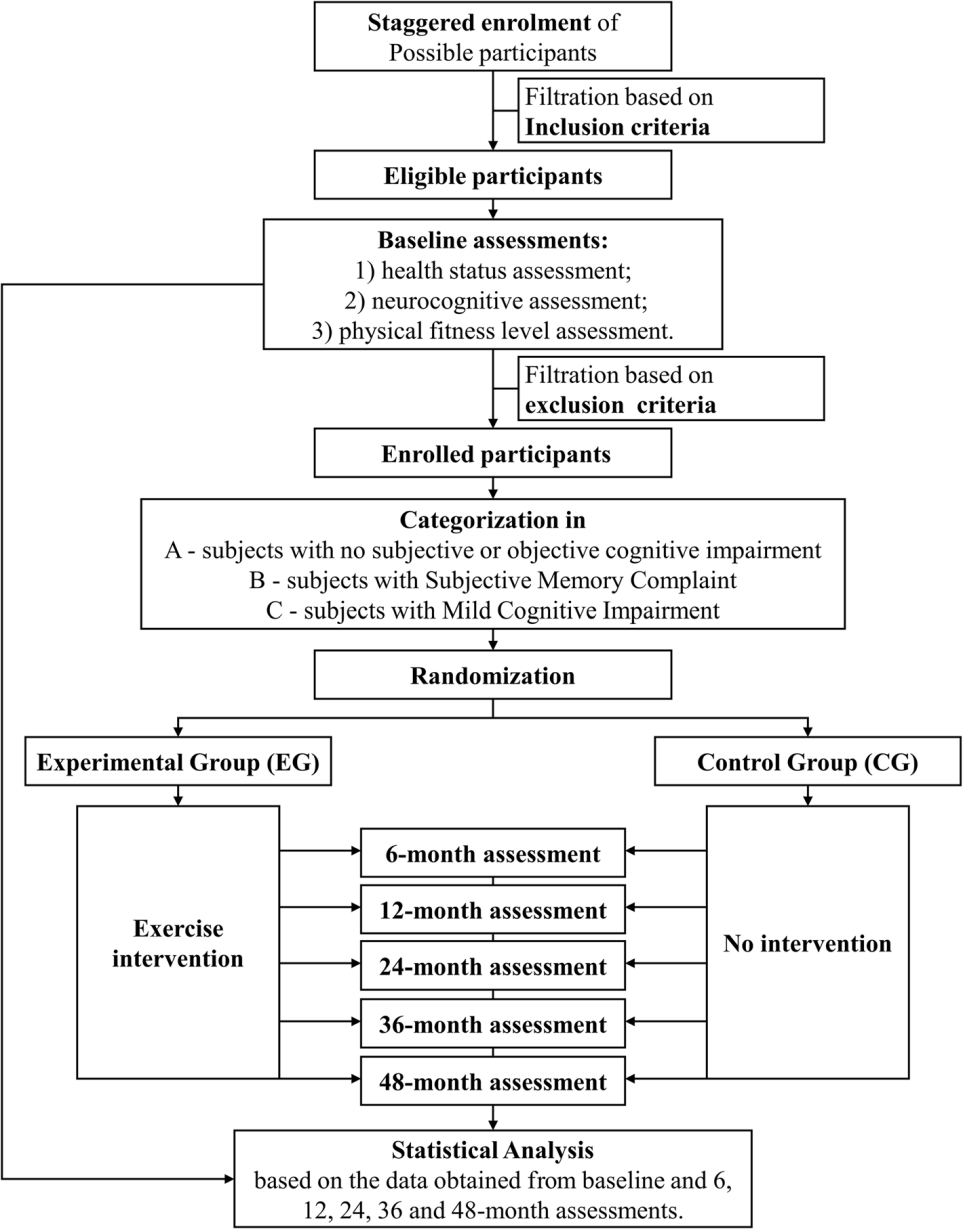
Flowchart of the study

This study was named EPD (Exercise for the Prevention of Dementia). EPD study is designed as a phase 3, parallel group, double-blind, 1:1 RCT, conducted using the CONSORT statement (http://www.consort-statement.org/) as a framework for the development of methodology. The paper also adheres to the main aspects of the SPIRIT guidelines for reporting clinical trial study protocols. The flowchart of the study is reported in Fig. 1.

### Participants

Community-dwelling volunteers aged ≥ 50 years, with sedentary or normally-active lifestyle in accordance with the definition of Pate et al. [14], are being recruited from community centers for elderly in Campobasso, Italy. Recruitment methods include advertising, study presentations at community center, and word of mouth. Staggered enrollment of participants and relative data collection started in May 2011 and the estimated completion date should be May 2019.

Exclusion criteria include: a) short version of Geriatric Depression Scale (GDS) score > 6 due to the presence of clinically significant depression [15]; b) alcohol intake of more than 4 units/day; c) medical conditions that compromise survival, such as metastatic cancer, or limit exercise practice, such as severe cardiac failure; d) diagnosis of dementia according to International Statistical Classification of Diseases, 10th Revision (ICD-10) [16]; e) Mini- Mental State Examination (MMSE) age-education-adjusted score of less than 24 [17]; f) Clinical Dementia Rating (CDR) scale score of 1 or more [18]; g) inability to walk for 6 min without assistance [19]; h) use of drugs or other substance, or presence of a medical condition influencing the cognitive function. Eligible subjects are being divided into 3 category groups: A - without subjective or objective cognitive impairment; B - with SMC; and C - with MCI. Subjects in the category group A show a score < 25 at Memory Complaint Questionnaire (MAC-Q) [20], and a normal performance at neuropsychological test battery. Participants in the category group B show normal performance at neuropsychological test battery, including Rey’s Auditory Verbal Learning Test (RAVLT) and Prose Memory Test (PMT) [21], but a score ≥ 25 at MAC-Q. Participants in the category group C meet the International Working Group (IWG) diagnostic criteria on MCI [22], and show MMSE score ≥ 24, CDR score = 0.5 and pathological age-sex-education-adjusted scores at least one test of neuropsychological battery. For more clarity, a schematic representation of the groups is reported in Table 1.

**Table 1:** Criteria for the participants’ categorization

Category groups	Criteria		
	MAC-Q score	Neuropsychological tests	MMSE and CDR scores
A Subjects without subjective or objective cognitive impairment	< 25	normal	• MMSE ≥24 • CDR = 0.0
B Subjects with SMC	≥25	normal	• MMSE ≥24 • CDR = 0.0
C Subjects with MCI	Any score	At least one pathological score	• MMSE ≥24 • CDR = 0.5

*SMC* Subjective Memory Complaints, *MCI* Mild Cognitive Impairment, *MAC-Q* Memory Complaint Questionnaire, *MMSE* Mini- Mental State Examination, *CDR* Clinical Dementia Rating

This study has been designed and it is being conducted in accordance with the principles of the Declaration of Helsinki. It has been approved by the Local Ethics Committee and the Agency for Public Health of Molise Region, and was registered at ClinicalTrials.gov (NCT02236416). A written informed consent is required for all participants and the corresponding personal data are treated according with the Italian Legislative Decree 30 June 2003, n. 196 “Code concerning the protection of personal data”. The administration of the informed contents was performed by the researchers of the study.

### Baseline and successive assessments

The potential eligible participants are invited at the Center for Research and Training in Medicine of Aging of the University of Molise to an in-person assessment that includes: 1) general conditions and health status, 2) neurocognitive performances, and 3) physical fitness levels. The three assessments are performed individually in 3 non-consecutive days. The baseline assessments were also used to screen the participants to exclude those that meet exclusion criteria, and to categorize them in A, B or C category groups. Successively, the assessment sessions are repeated at 6, 12, 24, 36 and 48 months to evaluate the differences among time-points.

#### General conditions and health status assessment

The following information are collected: socio-demographic data, that is age, gender, ethnicity, income, instruction level and marital status; behaviors (e.g. smoking, alcohol consumption); medical history and clinical conditions, evaluated with a pre-defined check-list; anthropometric measures, that is weight, height, body mass index, waist circumference; blood pressure (checked with calibrated mercury sphygmomanometers); blood sample analysis (for assessing the cardiovascular risk), that is glucose, urea nitrogen, creatinine, total cholesterol, HDL and LDL cholesterol, triglycerides, transaminases, and complete blood count; and medications used.

The blood samples are collected after 12 h of fasting, between 8 and 10 am, and they are all analyzed in the central laboratory of the Local Public Health Institute.

Finally, in accordance with the recommendations proposed by the American Heart Association [23], participants with intermediate or high cardiovascular risk, following the classification of the ACSM [24], have to perform an ergometric test, in order to evaluate their effective eligibility for the proposed procedures.

#### Neurocognitive assessment

To measure the main cognitive functions of each participant, a neuropsychological test battery, administered by trained assessors in blind mode, is used. The battery consists of the tests described below.

*MMSE* for global cognitive assessment [17], *Frontal Assessment Battery (FAB)* for frontal lobe functions [25]; *Rey’s Auditory Verbal Learning Test (RAVLT)* [26] and *Prose Memory Test (PMT)* for learning and verbal memory [27]; *Attentive Matrices Test* for selective visual attention [27]; *Raven’s Progressive Matrices* for problem solving ability [28]; *Stroop Color Word Interference Test* [29] and *Trail Making Test (TMT) version A and B* [30] for cognitive flexibility and ability to shifting; and *Copying of Drawings* with and without landmarks for visual-spatial ability [31].

Furthermore, 15-item *Geriatric Depression Scale (GDS)* [15] and the *Short-form Health Survey (SF-36)* [32] were used to assess symptoms of depression and life quality respectively. The *MAC-Q* [20] was used for the subjective assessment of the memory complaint. The tests of the neuropsychological battery are administered in a fixed order with alternative form [33].

#### Physical fitness level assessment

The level of physical activity of each participant is assessed with the Physical Activity scale for the Elderly (PASE) [34] that is a questionnaire consisting of 12 items quantifying physical activity performed during leisure, household, and/or occupational activities.

The overall physical condition is evaluated by the American Alliance for Health, Physical Education, Recreation, and Dance (AAHPERD) fitness battery [35], including the following tests: *Sit and Reach Test*, for measuring flexibility of the trunk/leg; *Agility Test*, for testing agility and dynamic balance; *Soda Pop Test*, for the assessment of the oculo-manul coordination; *Arm Curl Test*, that measures muscular strength/endurance of the upper body; *Six minutes walking test (*replacing the half-mile walking ability test*)* for the assessment of the cardiorespiratory fitness.

### Procedures

After the baseline assessments, the subjects in each category group (A, B and C) is identified by a progressive number and randomly assigned in a ratio of 1:1 to experimental or control group, using a list of random numbers generated by a statistical software (SPSS). The list was kept in a sealed envelope and a researcher, not directly involved in the recruitment and in the evaluation of the participants, assigned the allocation numbers.

#### Experimental group (EG)

Participants randomized to the experimental group are asked to participate in one-hour exercise classes, three times a week, for 48 months. The classes, with a maximum of 12 participants, are organized in the rooms of the University Sports Center of the University of Molise and are led by experienced instructors trained to supply the same program in each class. Exercise program is individualized based on physical function, physical fitness level, health status, and exercise responses.

Exercise intensity are measured according to the Borg’s Rating of Perceived Exertion (RPE), a 6-to-20 points scale [36] on which a score < 11 is considered an effort equal to a light exercise, 12–13 to a moderate exercise, and 14–17 to a vigorous exercise [37]. The RPE scales were extensively used and demonstrate moderate to strong validity compared with other measures of cardiorespiratory exercise intensity, such as percent of the maximal oxygen uptake (% VO_2max_), percent of the maximum heart rate (% HRmax), and blood lactate concentrations [37]. The training program and RPE system were chosen and designed to be easily reproducible at home, to promote a physical active life-style also after the end of the study.

The exercise programs offer a wide variety of exercises including aerobic, strengthening/endurance (resistance), and flexibility (stretching) exercises. Each session consists of a warm-up, aerobic, resistance, cool-down and stretching phase.

The *warm up phase* lasts 5–10 min and consists of light-to-moderate intensity (RPE 10–12) aerobic activity. During this phase, fitness ergometers, such as treadmill and/or stationary bicycle, or walking/jogging activities are used to activate the biggest muscular groups. Then, the *aerobic phase* lasting 15–20 min is performed by treadmill, stationary bike, arm-cycle ergometer and/or running. Duration and intensity of exercise are gradual over time, starting with multiple bouts (< 2 min) of light to moderate intensity (RPE max 12–13) exercises up to two 10-min bouts of moderate to vigorous exercise (RPE max 16–17), after several week of training. Subsequently, the *resistance phase,* lasting 20–25 min and involving the principal muscular groups of limbs and trunk, is carried out by bodyweight exercises (calisthenics) and dumbbells up to 2 kg. Duration and intensity are also progressive over time, starting with 2 series of 10 repetitions (RPE max 11–12) up to 6 series of 20 fast repetitions (RPE max 14–16), for each muscular group. Finally, the *stretching phase*, lasting less than 10 min, consists in flexibility exercises for each major muscle/tendon groups. Participants are invited to stretch to the point of feeling tightness or slight discomfort, hold a static stretch for 20–30 s and repeat the exercise 2–3 times for each muscle/tendon group.

The instructors monitor exercise intensity during all sessions taking care of the needs of the participants.

Participants with a number of absences > 50% are considered not adherent to the training.

#### Control group (CG)

Participants assigned to this group will be strongly encouraged to keep their usual life-style, or continue their usual physical activity, generally of low intensity, consisting in long walks, and/or stretching, toning and/or balance exercises, or posture education. Control subjects changing the PA intensity from light (RPE < 11) to moderate or vigorous (RPE > 12) are excluded from the group.

### Adverse effects

Participants are asked about the presence of any adverse effects, such as musculoskeletal pain or discomfort, at each exercise session, and are monitored for symptoms such as angina and shortness of breath during the exercise classes.

### Outcomes

The primary outcome of the study will be 48-month change in MMSE scores. Secondary outcome will be 48-month change in neuropsychological test scores, including FAB, RAVLT, PMT, Stroop Color Word Interference Test, Attentive Matrices, Raven’s Progressive Matrices, TMT, Copying of Drawings with and without landmarks, and in GDS, SF-36, MAC-Q, PASE and AAHPERD fitness scores. Another outcome measure will be a composite cognitive score, which will be calculated for assessing the overall changes in cognitive performance. Each cognitive score will be standardized into a z score, by subtracting the baseline mean and dividing by the baseline standard deviation of the group. Regarding tests with more than 1 part, the standardization will be performed for each individual part. All z scores will be then averaged to calculate the composite cognitive score at each of the five time points (baseline, and 12, 24, 36 and 48 months). A positive score will point out a gain in cognitive performance, while a negative score a loss.

### Sample size calculation

Sample size calculations were based on the findings of previous studies and were performed separately for each category group. For heathy group, considering that MMSE scores decline 0.3 points in 4 years, we considered 0.6 points the minimal clinically important difference and used a Standard Deviation (SD) of 1.2 [38]. For SMC group, since that annual decline in MMSE score vary between 0.46 and 1.32 and that SD may vary between 1.48 and 2.6 [39], we considered 1.3 points the minimal clinically important difference and used a SD of 2.6. For MCI group, considering that the annual decline in MMSE score vary between 0.1 and 1.1 [40] and that SD may vary between 0.3 and 3.2 [41], we considered 1.6 points the minimal clinically important difference and used a SD of 3.2. Considering the percentage of drop-outs in previous similar studies and the length of the present study, we assumed a 30% attrition rate. Using an α level of less than 0.05, 90 participants per arm group will guarantee a power of 0.80.

### Blinding

The staff involved in collection of outcome measures and the data analysis is blinded to randomization assignment. During each follow-up evaluation, participants and examiners cannot provide or request information about the exercise program that subjects are performing.

### Randomization

After the baseline assessment, the participants of each category groups A, B and C were randomly assigned to one of the two groups (CG and EG) by using 1:1 blind randomization. For the randomization, a scheme generated via a computerized random number generator was used.

### Statistical analysis

Data will be analysed using the SPSS statistical software package (IBM, v.20.0, Chicago, IL, USA). Variables will be examined for outliers and extreme values by means of box and normal quantile-quantile plots, Shapiro-Wilk’s and Kolmogorov-Smirnov’s tests. When normal distribution cannot be accepted, the transformed variable (square, square root, logarithmic, reciprocal of square root or reciprocal transformations) will be reviewed. For normally distributed continuous variables, arithmetic means and SDs will be calculated, while for not normally distributed continuous variables, median, 25th and 75th percentiles will be calculated, according to the shaped distributions of two groups, as assessed by visual inspection of population pyramids. Sampling homogeneity (or baseline differences between groups) will be examined by means of chi-square (χ^2^) for categorical variables, independent-samples t-test for normally distributed variables and Mann-Whitney U test for not normally distributed variables. Differences between groups (experimental group vs. control group) and changes in the cognitive performance during the trial will be evaluated by means of Repeated Measures Analysis of Covariance (RM-ANCOVA) in the intention to-treat and complete-case analyzes, or with Linear Mixed Model analysis (LMM). The evaluations in the different time-points will be considered as the within-subjects factor whereas the two groups will be considered as between factor. Age, education level and gender will be included as covariates. The assumptions of equality of variances and sphericity will be assessed by means of Levene’s test and Mauchly’s test, respectively. The Greenhouse-Geisser or the Huynh-Feldt correction will be applied if the assumption of sphericity will be violated. Post-hoc independent-samples t-test will be performed to detect significant differences among trial conditions.

A *p*-value < 0.05 will be considered statistically significant.

## Discussion

We present the design of the ongoing EPD Study. To the best of our knowledge, this RCT represents the first long-duration study analyzing the effects of a structured, individualized and multi-modal exercise programs in older adults with different risk of dementia. The hypothesis is that engaging in a supervised program of exercise, consisting of aerobic/resistance training performed for 60 min, 3 times a week for 48 months could be efficacious in averting or at least slowing the memory decline in subjects with different risk of dementia, from normal (with low risk), to SMC (with moderate risk), up to MCI (with high risk) subjects.

Results from the EPD trial will be reported according to the CONSORT guidelines (http://www.consort-statement.org/) to exceed the limits related to the poor methodological quality of many previous RCT. We intend to complete data collection in May 2019 to carry out a study with a larger population sample and to verify if the positive effect of exercise continues beyond the first 6–12 months, that usually is the maximal duration of exercise interventions in the previous studies [42].

The main strengths of this study are its randomized prospective design, the utilization of community-dwelling elderly people, the long period of intervention, and the adequate sample size. Other important merits of this study are the high reproducibility and the home-based nature of the intervention.

Furthermore, the proposed exercise program involves multiple components of fitness including aerobic, strength, balance and flexibility training, and such multimodal approach is expected to give the best results on cognitive function, compared to aerobic exercise alone, as suggested by previous study and meta-analysis [43–46].

Concerning the mechanism by which exercise could influence cognition, the actual evidences do not allow definitive answers. Several potential mechanisms could contribute to improve brain health and cognitive performance as suggested by Kennedy et al. [47]: promotion of cardiovascular health, production of Brain Derived Neurotrophic Factor (BDNF), improvement of insulin sensitivity, reduction of stress and inflammation. All these potential pathways can have a role by which exercise may maintain or improve cognitive functioning, especially in the context of the aging brain. The review of Kennedy et al. [47] also stated that a greater understanding of these mechanisms is necessary to provide potential biomarkers for investigating the efficacy of differing exercise regimes on cognitive outcomes. In fact, the absence of univocal biomarkers or parameters to assess effects of exercise on brain health represents another important problem in this particular field of research that make difficult the comparability of the different studies outputs. Another problem is the variability of the results obtained in the different study of literature: Landrigan et al., [48] in a recent meta-analysis, investigated the effect of resistance exercise on cognition, and they concluded that resistance training appears to have positive effects on cognition, but future researches are needed to determine why the effects are so variable. Finally, no evidences exist concerning the “optimal dose of exercise” in order to have benefit on cognition, and studies are needed comparing the effects of trainings with different session duration and frequency as suggested by Sanders et al. [49].

However, despite these difficulties, different authors supported the hypothesis that exercise can have an important role in promoting brain health [50]. The first important role can be the prevention of dementia onset due to the amelioration of several cardiovascular risk factors, such as diabetes, hypertension, obesity and depressive symptoms, which are recognized as a reversible risk factor for dementia [51]. The second role of the exercise is a direct effect on brain health: in fact, exercise appears to have a beneficial effect in preserving neurogenesis [52, 53], and favoring neuroplasticity [54]. Furthermore, beneficial effects of exercise were demonstrated on secondary dementia such as dementia inducted by Chronic Obstructive Pulmonary Disease [55].

Nevertheless, despite the increasing interest on the exercise effects on brain health, some authors claimed that the protective effects of exercise on dementia or cognitive impairment may be overvalued [56, 57]. Also, previous studies showed overall that physical fitness levels are not strongly correlated with cognitive performance [58]. This discrepancy in author opinions and results suggests that further high-quality studies, and interventional studies including long follow-up periods and high reproducible exercise interventions, are necessary.

The EPD trial will: (a) evaluate the effectiveness of multimodal exercise program of 48 months in improving neurocognitive functions in individuals aged 50 years or older at different risk of AD; (b) identify the category of subjects and the cognitive function that gets the best benefits from such modality of exercise; c) examine the possible mechanisms by which exercise improve cognition.

In conclusion, this study, if successful, may represent an affordable and safe method to demonstrate that exercise represents a protective tool to delay the onset of dementia and the cognitive impairment in older adults at risk, such as subjects with SMC or MCI. Results of this study are expected to have important public health implications and a profound impact on future AD prevalence.

## Data Availability

Not applicable.

## Abbreviations

AAHPERD: American Alliance for Health, Physical Education, Recreation, and Dance
AD: Alzheimer’s Disease
CDR: Clinical Dementia Rating
EPD: Exercise for Prevention of Dementia
FAB: Frontal Assessment Battery
GDS: Geriatric Depression Scale
MAC-Q: Memory Complaint Questionnaire
MCI: Mild Cognitive Impairment
MMSE: Mini- Mental State Examination
PASE: Physical Activity scale for the Elderly
PMT: Prose Memory Test
RAVLT: Rey’s Auditory Verbal Learning Test
RCT: Randomized Controlled Trial
SD: Standard Deviation
SF-36: Short-form Health Survey
SMC: Subjective Memory Complaints
TMT: Trail Making Test
